# Clay Improvement with Burned Olive Waste Ash

**DOI:** 10.1155/2013/127031

**Published:** 2013-03-20

**Authors:** Utkan Mutman

**Affiliations:** Department of Civil Engineering, Kocaeli University, 41380 Kocaeli, Turkey

## Abstract

Olive oil is concentrated in the Mediterranean basin countries. Since the olive oil industries are incriminated for a high quantity of pollution, it has become imperative to solve this problem by developing optimized systems for the treatment of olive oil wastes. This study proposes a solution to the problem. Burned olive waste ash is evaluated for using it as clay stabilizer. In a laboratory, bentonite clay is used to improve olive waste ash. Before the laboratory, the olive waste is burned at 550°C in the high temperature oven. The burned olive waste ash was added to bentonite clay with increasing 1% by weight from 1% to 10%. The study consisted of the following tests on samples treated with burned olive waste ash: Atterberg Limits, Standard Proctor Density, and Unconfined Compressive Strength Tests. The test results show promise for this material to be used as stabilizer and to solve many of the problems associated with its accumulation.

## 1. Introduction

Although olive trees are distributed over all continents, 97% of the world production of olive oil is concentrated in the Mediterranean basin countries: Spain, Portugal, Italy, Greece, Turkey, Tunisia, and Morocco [1]. Ironically, while the olive oil itself provides health during its consumption, its resulting byproducts (olive mill wastes during olive processing) represent a serious environmental threat, especially in the Mediterranean, Aegean, and Marmara regions that account for approximately 95% of worldwide olive oil production [2]. The safe disposal of these wastes is very crucial because of their polluting effects on soil and water. They are produced in large quantities in short periods of time and must be properly disposed of in order to avoid environmental risks [3]. Since the olive oil industries are incriminated for a high quantity of pollution, it has become imperative to solve this problem by developing optimized systems for the treatment of olive oil wastes. Among the several processes being used nowadays, the ones described are the following: bioremediation, thermal processes, evaporation, membrane processes, electrolysis, aerobic and anaerobic digestion, ozonation, coagulation/flocculation/precipitation, and distillation [4].

Olive waste is the by-product after olives have been pressed and olive oil extracted. Olive oil waste has always been one of the biggest problems associated with the industry. For a long time, the olive oil industry has been troubled with the disposal of their waste. The most important use of olive waste is for fuel owing to the extremely high cost of energy. Recently, there has been an increased rate of interest in olive cake residue by power stations for the high temperatures it generates with minimum ash. Some countries (Greece, Italy, Tunisia, and Turkey) plan to develop the uses for olive waste in energy producing. Some small coal power plant could be reset to burn olive waste. Olive oil residue can also be used in some construction applications. In America, the olive waste has been mixed with bitumen as a component of road construction material. Olive bricks, although lighter than that of traditional bricks, are also manufactured [5].

On the other hand, especially clayey soils required treatment. In this treatment, waste can be used. In this way, the waste is stabilized, and clayey soil is improved. This study evaluates the use of the burned olive waste ash as a soil stabilizer. In the literature, various additives such as lime, cement, and fly ash were used to stabilize expansive soils [5]. However, the literature survey reveals that there is a limited research carried out on the stabilization of expansive soils using the olive cake residue [6]. 

## 2. Materials and Methods

In this study, bentonite clay was used to improve. The bentonite was dried in an oven at 105 ± 5°C. Properties of the bentonite are given in Table 1. The olive waste, used in this study, was taken from the olive oil factory in Gemlik, Turkey. Olive waste was burned in a high temperature oven at 550°C about 1 hour. Then, the olive waste ash was sieved in 0.425 mm sieve and was used to pass through the sieve. The specific gravity of the burned olive waste is 1.48.

### 2.1. Test Procedure

The experiments were done by adding the olive waste ash at weight percentages of 0, 1, 2, 3, 4, 5, 6, 7, 8, 9, and 10% accordingly. When every sample was prepared, bentonite was initially mixed with the olive waste ash and was then kept under curing conditions for one hour for chemical process [7]. 

First, Atterberg Limit Tests were performed. The tests were conducted in accordance with ASTM 4318. Then Specific Gravity Tests were performed according to ASTM D854, and the Modify Proctor Tests were performed for determining optimum water content. The Modify Proctor Tests were conducted in accordance with ASTM D1557. 

Samples which were used at The Unconfined Compressive Strength Test were prepared at the optimum water content. Every ash weight percentage sample was cured for 0, 1, 7 and 28 days. After curing, The Unconfined Compressive Strength Test was performed. The Unconfined Compressive Strength Tests were conducted in accordance with ASTM D2166.

## 3. Results and Discussion

**Figure 1:**
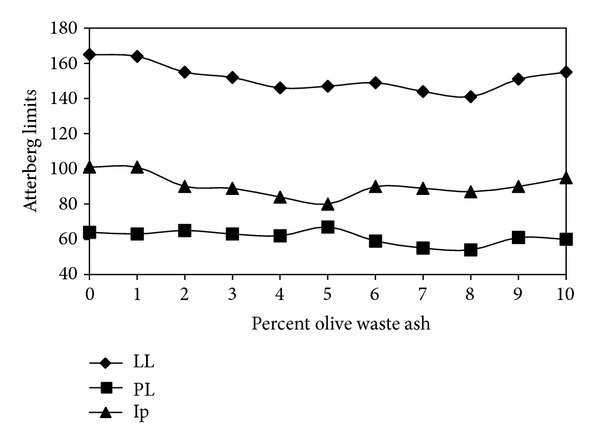
Effect of olive waste ash on the Atterberg limits.

Figure 1 shows the effect of burned olive waste ash on the Atterberg limits of the bentonite. Changing of olive waste ash in the bentonite causes a small change in the Atterberg limit values. The figure indicates that when the percent of olive waste ash increases, the Atterberg limit of the treated bentonite decreases. However, the plasticity index of the treated soils decreases with the increase in percent olive waste.

**Figure 2:**
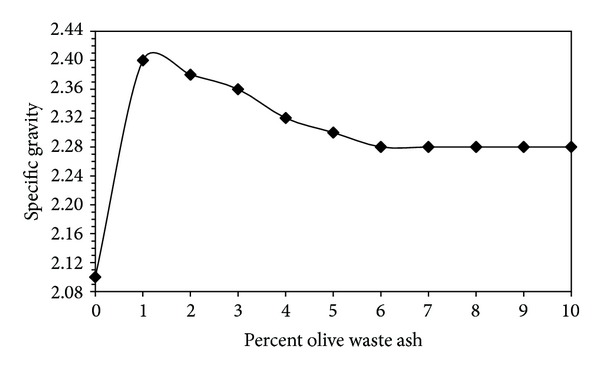
Effect of olive waste ash on the specific gravity.

The effect of burned olive waste ash on the specific gravity of the bentonite is showed in Figure 2. Rising amount of olive waste ash in the bentonite causes decrease in the specific gravity values. When 1% by weight the olive waste ash was added, specific gravity had maximum value. When the olive waste ash was added, specific gravity decreases. After the addition of 6% of the olive waste ash, specific gravities were constant. 

**Figure 3:**
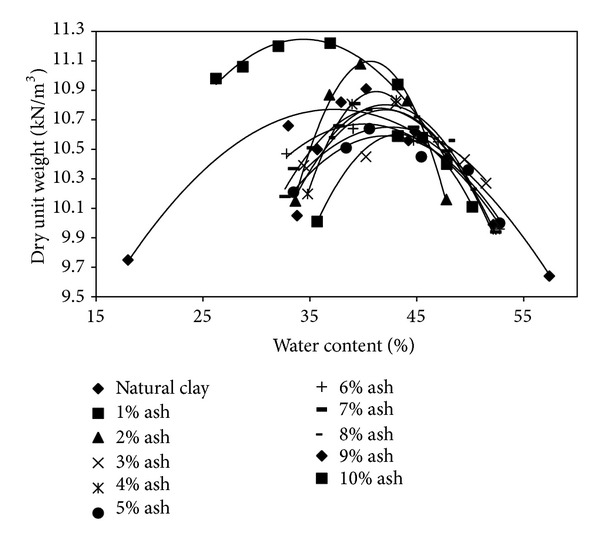
Compaction test results due to the addition of burned olive waste.

**Figure 4:**
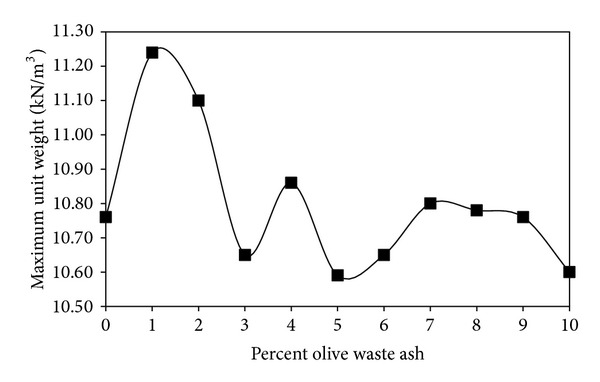
Effect of olive waste ash on the maximum dry unit weight.

Figure 3 shows the effect of olive waste on the compaction characteristics of the bentonite. Moisture-density relationships were based on the Harvard Miniature apparatus.  Figure 4 indicates that the highest maximum dry unit weight value was obtained by the addition of 1% by weight olive waste ash bentonite. The addition of higher percentages causes reducing the maximum dry unit weight. That is due to the fact that the olive waste has lighter particles than those of the bentonite. The lower specific gravity value of the olive waste ash reduces the mass of the soil for the same volume, resulting in a lower dry unit weight values [5].

**Figure 5:**
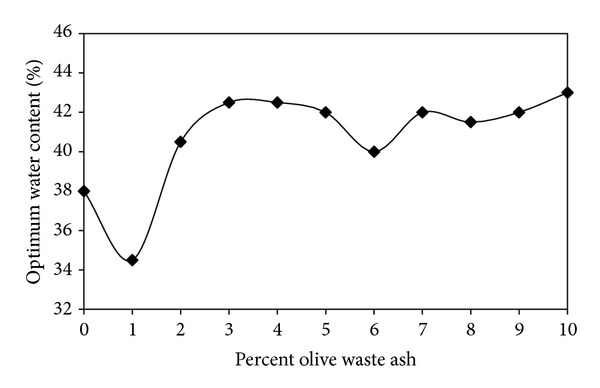
Effect of olive waste ash on the optimum water content.

On the other hand, Figure 5 indicates that the lowest optimum water content value was obtained for the addition of 1% by weight olive waste ash. The addition of higher percentages causes rising the optimum water content value.

**Figure 6:**
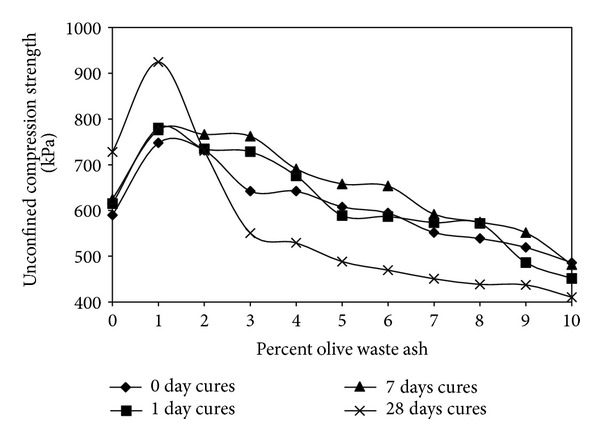
The effect of burned olive waste on the unconfined compressive strength of the treated soil.

Figure 6 shows the changing of unconfined compression strenght. It was noticed that the unconfined compressive strength of all specimens increased to its greatest value when the burned olive waste ash was added by 1% by weight. This can be explained that, as the maximum dry unit weight increases at 1% by weight, the soil becomes denser due to void filling by this material and consequently yields at a higher load. In spite of that, at higher percentages, by the reason of the fact that unit weight of ash is lower than bentonite, the maximum dry unit weight decreases. At the cure times of 0, 1, and 7 days, all specimens acted the same. At 9 the cure times of 28 days, unconfined compression strength decreases strongly when the addition of higher percentages.

Also, CaCO_3_ present in the burned olive waste has been converted to CaO and CO_2_, CaO, or Ca(OH)_2_. The addition of water can react with the amorphous silica remaining after burning the olive waste or clay in the soil to form calcium silicate hydrate, which is cementitious (or, in other words, the ash of the olive waste is pozzolanic). This would also contribute to the increase in the unconfined compression strength. The decrease of the unconfined compressive strength at a high percentage of burned olive waste is due to a decrease in the density and an increase of the noncohesive material in the samples [6].

## 4. Conclusion

The plasticity index of the treated soils decreases with the increase in percent olive waste.

When 1% by weight the olive waste ash was added, specific gravity had maximum value. To increase the percentage is caused to decrease specific gravity. 

The highest maximum dry unit weight value was obtained for the addition of 1% by weight olive waste ash. On the other hand, the lowest optimum water content value was obtained by the addition of 1% by weight olive waste ash.

The unconfined compressive strength of the all cure times of specimen is maximum value when the burned olive waste ash was added 1% by weight.

At the cure times of 0, 1, and 7 days, all specimens acted same. At the cure times of 28 days, unconfined compression strength decreases strongly when the addition of higher percentages.

## Figures and Tables

**Table 1:** Properties of the bentonite.

Chemical analysis	

L.o.I. (%)	7.50 ± 1.00
SiO_2_ (%)	71.00 ± 1.00
Al_2_O_3_ (%)	14.00 ± 1.00
Fe_2_O_3_ (%)	0.70 ± 0.10
TiO_2_ (%)	0.05 ± 0.01
CaO (%)	1.10 ± 0.30
MgO (%)	3.20 ± 0.20
Na_2_O (%)	0.25 ± 0.05
K_2_O (%)	1.00 ± 0.10

Mineralogical analysis	

Montmorillonite (%)	80
Cristobalite-opal C (%)	17
K-feldspar (%)	3
Plagioclase (%)	Trace

Properties	

Cation exchange capacity (meq/100 gr)	85.0 ± 5.0
CaCO_3_ (%)	0
Swelling (mL/2 gr)	8.0 ± 2.0
Sedimentation (72 hours) (mL)	10
Sintering point (°C)	1200
Bulk density (gr/lt)	800 ± 30
Clumping test	Positive
Clump weight (gr)	55 ± 5
Water absorbtion (%)	90 ± 5
Water absorbtion duration (sec)	max. 65
NH_3_ adsorption (ppm)	40
Bleaching—original(Tonsil equivalent)	0.6
Bleaching—Acid activated (Tonsil equivalent)	0.7
pH (8% solid)	8.5
Grit content (+75 *μ*m) (%)	<4
Color	White
Lightness	93 ± 1
Moisture (%)	<30
